# The recommended patient syndrome: Charting new frontiers

**DOI:** 10.5339/qmj.2024.68

**Published:** 2024-10-28

**Authors:** Ibraheem M. Alkhawaldeh, Jaber H. Jaradat, Raghad Amro, Abdullah Ashraf Hamad, Hashem Abu Serhan

**Affiliations:** 1 Faculty of Medicine, Mutah University, Al Karak, Jordan; 2 Faculty of Medicine, Menoufia University, Menoufia, Egypt; 3 Medical Research Group of Egypt, Negida Academy, Arlington, MA, USA; 4 Department of Ophthalmology, Hamad Medical Corporation, Doha, Qatar *Email: HAbuserhan@hamad.qa

**Keywords:** Recommended patient syndrome, RPS syndrome, very important syndrome, VIP syndrome, review

## Abstract

**Introduction:**

Very important person (VIP) syndrome, or recommended patient syndrome (RPS), is a phenomenon in healthcare where well-intentioned efforts to enhance care inadvertently result in harm. Celebrities, physicians, and political leaders often receive preferential treatment, leading to potentially unnecessary interventions. This review delves into its causes, manifestations, consequences, real-life instances, prevention, and management, considering the shifting paradigm of recent advances in the medical field.

**Methods:**

We conducted a qualitative and critical review by searching databases such as PubMed, Scopus, and Google Scholar, as well as online news outlets, exploring available data and literature, including journal articles and news articles published at any time on VIP syndrome.

**Results:**

Twenty articles were found relevant and analyzed. Manifestations of VIP syndrome range from over- to under-treatment, disrupting established healthcare systems. Consequences encompass increased costs, heightened risks, and diminished satisfaction for patients and healthcare teams. Real-life instances, exemplified by Michael Jackson’s case, highlight unintended complications. Prevention strategies advocate transparent resource allocation and adherence to established guidelines. A written VIP patient management plan, involving the hospital’s command center, security, and press spokesperson, is crucial. Proposed directives underscore the importance of valuing medical skills, teamwork, effective communication, and resisting external pressures.

**Conclusion:**

This short communication underscores the necessity of systematically addressing VIP syndrome to ensure fair, ethical, and optimal healthcare delivery. By addressing this issue in an organized way, healthcare providers can work towards treating all patients equally, following ethical guidelines, and providing the best possible care to everyone, regardless of their status or influence. Future research should focus on developing standardized protocols for managing VIP patients, incorporating ethical considerations and evidence-based practices.

## 1. Introduction

Very important person (VIP) syndrome, or recommended patient syndrome (RPS), is a paradoxical phenomenon in healthcare where patients’ connections with healthcare providers, social status, or political positions lead to receiving preferential treatment, often compromising optimal care and may result in unnecessary or harmful medical interventions.^1-4^ The terms RPS and VIP syndrome, coined by Dr. Walter Weintraub and Winston Churchill, respectively, reflect a consequence of good intentions that contradictorily undermine optimal treatment for individuals receiving preferential treatment in the healthcare setting.^2,3^ The VIP term typically implies images of celebrities; however, this is not the case for VIP syndrome, where it includes any individual receiving “special” treatment or creating tension for the healthcare providers to offer “special” care. Physicians, healthcare workers, celebrities, political leaders, hospital staff, and their families, recommended patients of influential figures, financial contributors, and others are all considered VIPs.^2^

As suggested by Sanz Rubiales et al.,^6^ the emergence of VIP syndrome in clinical practice can be attributed to various factors, including (a) patients’ preferences for a prestigious physician; (b) inadequate space and time for consultation; (c) incomplete clinical records; (d) incomplete or excessive use of diagnostic tools; (e) overtreatment in the fashion of “more is better than less.”^5-6^ The hierarchical reversal in decision-making with senior staff complying with patient demands can undermine the authority of junior staff members.^7^ Allen-Dicker et al. reported that 67% of the physicians treating VIP patients feel scrutinized and under immense external pressure about the possible outcomes of their patients. These physicians do not act according to medical reasoning, as 56% of the physicians agreed to the demands of their patients regardless of whether they were relevant or not.^7,8^ Regardless of the reason, by making healthcare teams feel scrutinized, these patients are at increased risk of poor medical treatment outcomes.^7^ The inefficient use of healthcare resources is another factor, with providers ordering unnecessary tests or treatments to maintain relationships or gain favor, which usually leads to a long hospital stay and harmful side effects, such as exposure to unnecessary amounts of radiation where physical findings or less radiation can be utilized.^7^

A special form of VIP syndrome is seen when physicians become patients, with reported cases of the physician being consulted on their case, or when physicians complain of isolation and deprivation of necessary information about the course of their illness, as the attending physician wrongly assumes that the physician-patient needs less explanation about their illness and the caregiving routine considering their knowledge.^2,9^ Moreover, 81% of physicians reported that physician patients received privileges over other patients, including faster access to care and longer hospital stays due to their contacts.^10^ Having a physician-patient was significantly associated with negative feelings experienced by the physician treating them, as 52% of the interviewed physicians experienced stress and feeling judged when treating a physician,^10^ because physicians may have another opinion on their treatment course and may interfere with their management, creating more tension for the treating physician.^7^

## 2. Manifestations and Consequences

**Figure 1.**
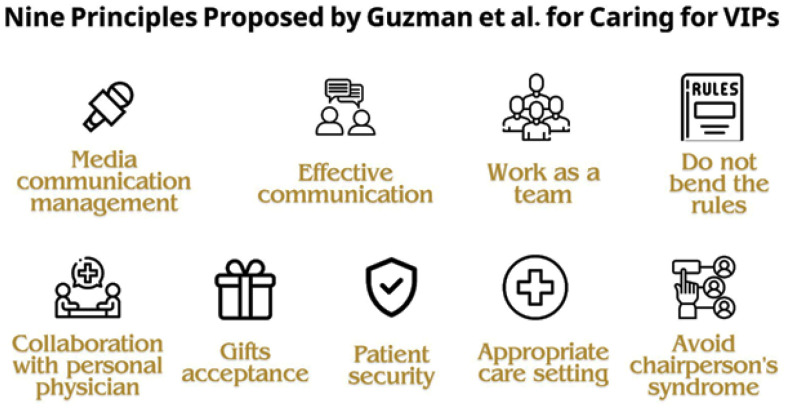
Summarization of the potential consequences of VIP syndrome on patient and healthcare system.^4,11,16^

The consequences of VIP syndrome are multifaceted, impacting patients, healthcare teams, and the healthcare system. These effects can be observed in various ways (Figure 1), including:

1. Unnecessary tests and follow-ups: VIP patients may undergo unnecessary tests and frequent follow-ups, with an inclination toward monitoring minor abnormalities. This can lead to avoidable side effects and pain.^11^ Conversely, fewer tests or therapeutic maneuvers to spare VIPs discomfort may also occur, potentially resulting in missed diagnoses.^11^ Both situations compromise the quality of care provided and disrupt the established healthcare system.
2. Impact on patients and healthcare teams: VIP syndrome has consequences for patients and physicians, including increased healthcare costs, elevated risk of adverse events, and diminished satisfaction. The bad experiences of both patients and healthcare teams result in suboptimal treatment, disagreement among team members, and patient non-compliance. Paradoxically, the VIP status intended for better healthcare becomes a risk factor for complications and treatment failures.^4,11^
3. Premature discharge and isolation: A shared desire among healthcare teams to discharge VIP patients quickly may result in premature discharge and inadequate recovery. Additionally, healthcare staff may seek excuses to minimize contact due to patient demands, contributing to the isolation of VIP patients. This perpetuates a cycle of poor care, increased patient anxiety, and additional pressure on the healthcare team.^16^
4. Erosion of trust and inefficient resource allocation: VIP syndrome contributes to the erosion of trust within the healthcare system. When preferential treatment is given to certain individuals, it affects the fairness and equity of resource allocation. This can lead to inefficient use of healthcare resources, as they may be directed towards VIP patients rather than being distributed based on medical needs.^16^

## 3. Real Life Instances

There have been reported cases of unintended complications and preferential treatment occurring to VIP patients, ranging from celebrities and influential figures to physicians and their families. In one reported case, a 55-year-old physician relative presented with simple symptoms suggesting acute diarrheal disease. However, excessive tests were performed, and the patient suffered from unusual complications, including contrast allergy, massive atelectasis, and post-traumatic stress.^4^ Another infamous example is the death of the pop singer Michael Jackson, which many attribute to VIP syndrome, as the singer’s doctor administered a combination of propofol, midazolam, and lorazepam simultaneously without proper monitoring, and it was consequently ruled as a homicide.^12^

The undesirable consequences of VIP syndrome are not limited to complications and treatment failures after managing the VIPs; they also extend to unfair allocation of healthcare resources and preferential access to experimental treatments without meeting the eligibility criteria of clinical trials. Reports of unfair allocation of COVID-19 testing and vaccines were published during the pandemic when it was suggested that influential figures, including politicians and celebrities, were offered testing and vaccinations ahead of the public owing to their status and connections.^13^ Former United States President Donald Trump’s case highlights the VIP syndrome concerning access to experimental treatments. Trump gained access to REGEN-COV, a mixture of Casirivimab and Imdevimab, which are neutralizing antibodies against the spike protein of SARS-CoV-2 that work effectively in reducing the viral load of the virus. The Former President received treatment while it was still under development and before it received emergency use authorization in November 2020,^14^ which raises concerns about the ethicality of such a decision that was most likely taken due to Trump’s VIP status.^15^ Moreover, it was reported that after being infected with SARS-CoV-2, Trump received a course of steroids that are typically reserved for severe cases of the disease, further indicating VIP syndrome.

Recently, the increasing prominence of celebrities as social media influencers has the potential to impact the VIP patient syndrome. This phenomenon can lead to alterations in behaviors and clinical practices triggered by a patient’s unique social or political standing, resulting in a cycle of heightened VIP expectations and healthcare staff disengagement—an aspect that should be anticipated in the future.^1^

## 4. Prevention

With such consequences of VIP syndrome, prevention should be of utmost importance and priority in all healthcare facilities, and several strategies must be implemented to minimize its occurrence. Some of the strategies that may be used include instituting oversight committees or ethics boards within healthcare facilities to ensure adherence to established guidelines and to review potential cases of preferential treatment. The hierarchical system of physicians caring for the VIP patient should be no different from the one followed for other patients, with one attending physician leading the case and junior staff closely monitoring the patient and reporting to the attending physician as they have more frequent contact with the patient.^16^

Most importantly, to prevent and reverse the damages of VIP syndrome, the attending physician must take command and follow the rules of the healthcare facility, ensure the privacy of the patient, limit the number of visitors who may try to influence the plan of care, and explain clearly to the patient and all the staff involved in the case that the care will be identical to the care of all other patients who have the same condition.^11^ Moreover, healthcare facilities should adopt transparent systems for resource allocation, ensuring that medical resources, including staff, equipment, and facilities, are allocated based on medical needs rather than status or influence.

VIP healthcare services present significant ethical issues, particularly concerning fairness and equal access to care. This system allows wealth and status to influence the quality of treatment, deepening existing social disparities, especially across racial and socioeconomic lines. It fosters a hierarchy in healthcare where the affluent receive better care, potentially to the detriment of those with fewer resources who endure longer wait times. Ethically, this approach compromises principles of fairness and justice, implying that financial status dictates a person’s worth, which opposes the Alma-Ata Declaration’s view of healthcare as a fundamental human right. Moreover, the claim that VIP services help fund care for low-income patients lacks substantial evidence, with little indication that profits are used to support marginalized communities.^17^ This model risks compromising healthcare providers’ ethical duties, diminishing trust among vulnerable groups. Ultimately, healthcare systems should focus on providing equal care to all, with access based on medical necessity rather than wealth.^17^

Staff allocation is especially important, as it may be affected by patients exhibiting chairperson’s syndrome, which occurs when the patient or their family requests to be cared for by the department’s chairperson, assuming that they would be the best doctor,^1^ which in many cases might not be true considering that most senior physicians have become more involved with administrative and managerial tasks than hands-on interaction with patients. Chairperson’s syndrome is prevalent among physicians’ families as physicians’ children saw significantly fewer junior physicians in the emergency department when compared to other children.^18^

## 5. Management of VIP Patients

Caring for the VIP patients should ideally come with a written plan, one that involves the hospital’s command center, security personnel, and press spokesperson, and should include details of which staff to attend to the VIP, especially if they visit the emergency department, who to be notified and in what sequence, a plan for ensuring the security and privacy of the patient, if required, and what necessary information might be given to the press.^19^ This coordinated approach helps streamline the patient’s care and maintain a structured environment. For instance, in one reported case, a 52-year-old princess from the United Arab Emirates refused to be taken care of by female physicians, so it was consequently planned for her to be taken care of by male physicians, which calmed the patient and reduced her anger previously directed at the hospital.^2^

As all physicians worldwide need guidelines to outline how to manage VIP patients and prevent the negative consequences of VIP syndrome, Mariano et al. proposed three directives for managing VIP patients: V: Vow to value your medical skills and judgment; I: Intend to command the medical aspects of the situation; and P: Practice medicine in the same way for all your patients.^20^

**Figure 2.**
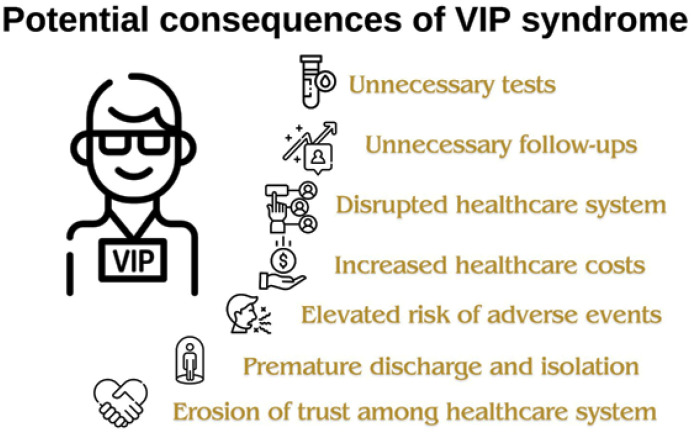
Principles proposed by Guzman et al. for caring for VIP patients.^1^

Moreover, Guzman et al. proposed nine principles for caring for VIPs (Figure 2).^1^

(a) Do not bend the rules: Despite the pressure created by the VIP patients, physicians should not change the time-tested rules and guidelines placed by the hospital.
(b) Work as a team: There should be one physician who is the main physician attending the case, but all other consultants who may be involved in the case should work together to avoid uncoordinated care.
(c) Effective communication: Physicians caring for the VIP should communicate well with the patient, their family, and other physicians.
(d) Media communication management: Only information that the patient has consented to in advance should be released to the media, and any coverage should be carefully coordinated with the hospital’s public relations department.
(e) Avoid chairperson’s syndrome: Staff allocation should be based on the patient’s medical condition rather than their VIP status.
(f) Appropriate care setting: Care should occur where it is most appropriate, which means that if the condition calls for care in the intensive care unit, the desire to give the VIP patient more privacy by placing them in another unit should be resisted.
(g) Patient security: Because the media frequently follow high-profile patients, precautions should be taken to protect their privacy, safety, and security.
(h) Gifts acceptance: Be careful about accepting or declining gifts; it is advisable to decline gifts during the patient’s hospital stay, but after hospitalization is over, you are welcome to accept a fair and appropriate gift.
(i) Collaboration with personal physician: If the VIP patient has a personal physician or physician, it is best to seek advice and express appreciation for them. However, it must be made clear that the attending physician will be the one ordering all diagnostics and treatments during the patient’s hospital stay and that the decision they and their patient can agree upon will ultimately be the decision to be followed when it comes to the patient’s care.

## 6. Conclusion

VIP syndrome refers to the special treatment and attention given to patients with social, political, economic, or family ties to healthcare professionals. While these patients may receive preferential care, this can lead to negative outcomes, including unnecessary tests and treatments, increased risks of complications, and unfair allocation of healthcare resources. Immediate action is needed to limit the spread of this syndrome and protect patients against its consequences. Further research is needed to highlight and assess the prevalence of this syndrome and provide common guidelines to avoid this syndrome.

## Ethics Approval

The study followed the principles of the Helsinki Declaration, and no institutional review board (IRB) approval was necessary because it did not involve human subjects.

## Conflicts of Interest Statement

The authors have no relevant financial or non-financial interests to disclose.

## Authors’ Contributions

IA and HS: Conceptualization; Designing the manuscript; Literature search; Writing an original draft; and Final review. JJ: Designing the manuscript; Literature search; Writing an original draft. RA: Literature search; Writing an original draft. AH: Creating manuscript figures; Final review and editing. All authors read and approved the final content.
